# Sedentary lifestyle with increased risk of obesity in urban adult academic professionals: an epidemiological study in West Bengal, India

**DOI:** 10.1038/s41598-023-31977-y

**Published:** 2023-03-25

**Authors:** Sunandini Ghosh, Manabi Paul, Kousik Kumar Mondal, Sandip Bhattacharjee, Pritha Bhattacharjee

**Affiliations:** 1 Environmental Epigenomics Lab, Department of Environmental Science, University of Calcutta, 35, Ballygunge Circular Road, Kolkata, West Bengal 700019 India; 2 Department of Zoology, University of Calcutta, 35, Ballygunge Circular Road, Kolkata, West Bengal 700019 India; 3 Department of Zoology, Mugberia Gangadhar Mahavidyalaya, Bhupati Nagar, West Bengal, Purba Medinipur, 721425 India; 4 Health Management, Siemens Limited, Mumbai, India

**Keywords:** Diseases, Health occupations

## Abstract

Ectopic fat deposition is more strongly associated with obesity-related health problems including type 2 diabetes mellitus (T2DM), cardiovascular diseases (CVD), hypothyroidism, arthritis, etc. Our study aimed at identifying the cumulative role of several risk factors in developing obesity and the role of ectopic fat (visceral fat) in predicting cardiovascular disease risk in varied age groups among urban adult academic professionals in West Bengal. 650 adults (Male = 456; Female = 194) associated with the academic job (age 20–65 years) in urban West Bengal were randomly selected for anthropometric, blood biochemical, and questionnaire-based analyses. Body Mass Index and Visceral Fat% exhibited comparable association with all the other anthropometric parameters (e.g. Whole body Subcutaneous fat%: male-Linear Regression Comparison: *F* = 11.68; *P* < 0.001; female-*F* = 6.11; *P* < 0.01). Therefore, VF% acts as a risk factor alongside BMI in instances where BMI fails alone. The presence of T2DM, hypertension, and hypothyroidism in the case groups confirmed their obesity-associated longitudinal pattern of inheritance. Unhealthy diet pattern indicates improper liver function, vitamin D deficiency, and increased erythrocytic inflammation. An overall sedentary lifestyle with parental history of obesity was found to be significant in the longitudinal transmission of the disease.

## Introduction

Obesity, commonly caused due to abnormal fat deposition, is primarily measured by increased body mass index (BMI)^1^. This global pandemic has increased concern regardless of the economic condition of a country^2^. In 2015, about 30% of the world’s total population, including 107.7 million children and 603.7 million adults were found to have obesity worldwide^3^. It can also be predicted that in 2030, the global population of overweight and obesity will increase to 2.16 billion and 1.12 billion, respectively^4^. In India, the National Family Health Survey-4 (NFHS) reported18.9% of men as overweight, including 26.6% of urban and 14.3% of rural men, whereas 20.6% of all women were found to be overweight, accounting for 31.3% of urban and 15.0% rural women^5^. Furthermore, a study also indicates that the rate of increase in obesity becomes highest in early adulthood^6^.

The obesity-associated health problems refer to complex metabolic diseases, also known as lifestyle diseases, including diabetes, cardiovascular diseases, arthritis, polycystic ovarian diseases, etc. that are intimately associated with obesity^7,8^. It has become necessary to identify and estimate the factors associated with increasing co-morbidities leading to obesity to curb the exponential growth curve. Although fat accumulation mostly occurs in subcutaneous adipocytes, the deposition has also been found in ectopic sites such as the visceral area, liver, muscle, heart, and pancreas^9^. Increasing age influences the distribution of adipose, shifting it from subcutaneous depots to intra-abdominal and ectopic fat deposition^10^. BMI is considered to be the most common yardstick to measure obesity worldwide, yet as it is not capable of differentiating between body fat and muscle mass, it fails to be a reliable predictor of disease risk^11^. Therefore, body composition monitoring can become crucial to identify the visceral fat percentage (VF%) which signifies central obesity. Accumulated fat like epicardial and extra-pericardial are responsible for CVD risk and correlate well with increased VF%. Moreover, VF has been related to both cardiovascular and metabolic dysfunction^12^. In Asian Indians, intra-abdominal VF accumulation causes central obesity rather than a generalized one^13^.

The environment plays a complex interactive role along with genetic imprints. Modifiable factors like lack of physical activity, calorie-rich diet, sleeping disorders, etc. are one of the major game-changers of obesity, while the other one is parental history^14,15^. Poor dietary patterns coupled with increased leisure time including television watching and minuscule physical activity owing to technological development (using elevators, digitalization of manual labor, etc.) and selection of residential environment (metropolitan cities with higher facilities to minimize physical movement) accounts for increasing obesity^16^. Studies on children and adolescents indicate that not only maternal but the paternal history of obesity and associated co-morbidities also account for the offspring’s health^17,18^. Even though limited information is available on central obesity among adults in West Bengal, however, there seems that the frequency of overweight among the studied Bengali population is at an alarming stage^19^. A comparative study of 224 urban and 224 rural Bengali adults reported the prevalence of metabolic syndromes to be highly significant in both sexes in the urban population^20^. Diet, along with physical activity, plays an important role in lifestyle. Urbanization, advertisement, and easy access to supermarkets have westernized the dietary pattern in modern urban India^21^. The industrialization of the agricultural sector over time has increased the chemical burden on the neutral ecosystem which has affected the healthier food habit of the Bengali population^22^. A sedentary lifestyle involving a lack of physical exercise is prominent in professions like academics, judicial, information technology, etc. Physical activity is the only established modifiable variable that can be considered to regulate the total energy expenditure, thereby directly influencing the obesity status of a nation^23^.

The lack of any previously published data has driven an utmost urge to analyze the present obesity status of an occupation-oriented sedentary lifestyle in academic professionals. Hence our study focussed on the assessment of the cumulative role played by several risk factors on the vulnerable unique urban adult population in West Bengal of different age groups with similar food habits and sedentary lifestyles.

## Methods

### Study area

Our study was conducted between September 2017 and September 2018. We selected three campuses of the University of Calcutta, West Bengal, India, as the study areas based on the participants, viz. the urban academic professionals (Supplementary Fig. 1). Epidemiological surveys were conducted during September, October, and November 2017 in three different campuses. Sample collections were done in December 2017; while data analyses were conducted in 2018.

### Study participants

Random sample collection was conducted in all three study sites. Sample size calculation following Cochran’s Formula^24^, indicated a required minimum sample size of 281 assuming the prevalence of obesity to be 24% at sample collection time^25^ with a 95% confidence interval to achieve the power of 80%. Consecutive sampling resulted in the voluntary participation of a total of 671 individuals including both male and female college students, research fellows, faculties, and non-teaching staff of different age groups ranging from 20 to 65 years. The male–female ratio was found to be approx. 2:1.

### Data collection

The data collected in the three respective study sites involved three phases. Phase I consisted of socio-demographic data collection. The participants were interviewed privately using a predesigned pretested questionnaire only after receiving the University Ethical Clearance and their written informed consent. All the methods were carried out in accordance with relevant guidelines and regulations approved by Institutional Ethical Committee of the University of Calcutta (Ref No. 003/17-18/1676). Participation was voluntary. Phase I was followed by Phase II involving anthropometric measurements and Phase III including blood biochemical analysis.

### Anthropometric measurement

Anthropometric measurements were initiated by recording the height of the individual (without shoes) in centimeters. The bioelectrical Impedance Analysis (BIA) technique was applied to the other anthropometric parameters. For ideal weight management and a more accurate and precise body composition analysis, a full Body Sensing Technology Karada Scan Body Composition Monitor (Omron HBF-375, Kyoto, Japan) was used following the detailed mechanism^26^ which measured body composition such as weight (in Kg), total body fat percentage, visceral fat percentage (VF%), subcutaneous fat (WbSb%) and skeletal muscle percentage (WbSk%) and body mass index (BMI) (in kg/m^2^). Systolic and diastolic blood pressures (SBP and DBP respectively) were measured using an automatic digital blood pressure monitor (Omron HEM 7120, Kyoto, Japan) according to the protocol explained previously^27^. The data collection was followed by the calculation of the mean arterial pressure (MAP) (in mm/Hg) using the standard formula i.e., MAP = DBP + 1/3(SBP − DBP). Out of the total 671 participants, 650 (456 male and 194 female) were shortlisted for our statistical analysis based on individuals without obesity (BMI: 18.5–24.9 kg/m^2^) and with overweight and obesity (BMI ≥ 25 kg/m^2^). We excluded the under-aged (< 20 years), underweight (BMI < 18.5 kg/m^2^), and pregnant female participants. The total 650 candidates were arranged into two separate groups according to their age, Group I (age < 35) years and Group II (age ≥ 35 years). Both of these groups were further classified into two subgroups based on their sex (male and female) i.e., Group I male, Group I female, Group II male, and Group II female. Furthermore, each subgroup was classified into two case–control groups based on BMI (‘case’ referring to overweight-obese and ‘control’ referring to non-obese healthy adults) viz. Group I case male (n = 51), Group I case female (n = 65), Group I control male (n = 56) and Group I control female (n = 60); Group II case male (n = 190), Group II case female (n = 50), Group II control male (n = 159) and Group II control female (n = 19) (Supplementary Table 1a). Confidentiality of the data was maintained throughout.

### Biochemical analysis

100 age-sexes matched overweight and obese individuals (50 male and 50 female) voluntarily registered for the biochemical analysis, of which only 25 male and 20 female representatives finally participated. 5 ml of blood was collected from each subject in (EDTA) coated vacutainer (BD Pharmaceuticals Pvt. Ltd., West Bengal, India) and outsourced to the pathological laboratory (Thyrocare Technologies Limited, Mumbai, India), for analysis of 48 biochemical parameters (Supplementary Table 3).

### Statistical analysis

All the demographic data were expressed as Mean ± SD. The anthropometric data were recorded and maintained using Microsoft Excel 2010. Statistical analyses were performed between all the subgroups in a case–control pattern. Linear regression, comparative linear regression, and 2-tailed unpaired t-test were done using Microsoft Excel (Washington, USA) and StatistiXL (Version 2.0, Broadway-Nedlands, Australia). The study was conducted following the STROBE checklist.

### Ethics guidelines

The authors confirm that all the experimental methods were carried out in accordance with relevant guidelines and regulations by the institutional ethical committee of the University of Calcutta (Ref No. 003/17-18/1676).

### Ethics approval

The study was approved by Institutional Ethical Committee of the University of Calcutta (Ref No. 003/17-18/1676).

## Result

### The anthropometric study

In our study design, 35.74% of the total study participants were aged < 35 years (Group I), and 64.25% were aged ≥ 35 years (Group II). The case–control (i.e. overweight-obese and non-obese normal weight) distribution pattern accounted for 54.76% and 45.23% respectively. Supplementary Table 1a represented the distribution details, mean age, and BMI of the subcategories of our studied population. The demographic details (Supplementary Table 1b) were not significantly different in the subgroups to be considered for causing any major disease manifestation.

In Group I, 96.42% of the male control participants, showed VF% ≤ 9^1^. In contrast, the rest had higher VF% despite having a normal BMI. However, for the case group of males, 86.27% revealed a VF% > 9. Unlike the males, 100% of the control females, as well as 66.15% of case females, showed VF% ≤ 9.

In Group II, 36% of the control male population reflected VF% > 9, in contrast to the case population (where 100% showed VF% > 9). Whereas for females, 0% of the control population showed VF% > 9 while, 68% of the case population had VF% > 9.

The 2-tailed unpaired t-Test showed that there was a significant difference between the male and female groups concerning BMI (*T* = 2.62, *DF*_*1,2*_ = 193,455, *P* < 0.05), VF% (*T* = 9.14, *DF*_*1,2*_ = 193,455, *P* < 0.001), WbSb% (*T* = 27.51, *DF*_*1,2*_ = 193,455, *P* < 0.001), WbSk% (*T* = 23.5, *DF*_*1,2*_ = 193,455, *P* < 0.001), SBP (*T* = 10.72, *DF*_*1,2*_ = 193,455, *P* < 0.001), DBP (*T* = 4.66, *DF*_*1,2*_ = 193,455, *P* < 0.001) and MAP (*T* = 8.13, *DF*_*1,2*_ = 193,455, *P* < 0.001).

#### Comparative association between VF% and WbSb%; BMI and WbSb%

**Figure 1:**
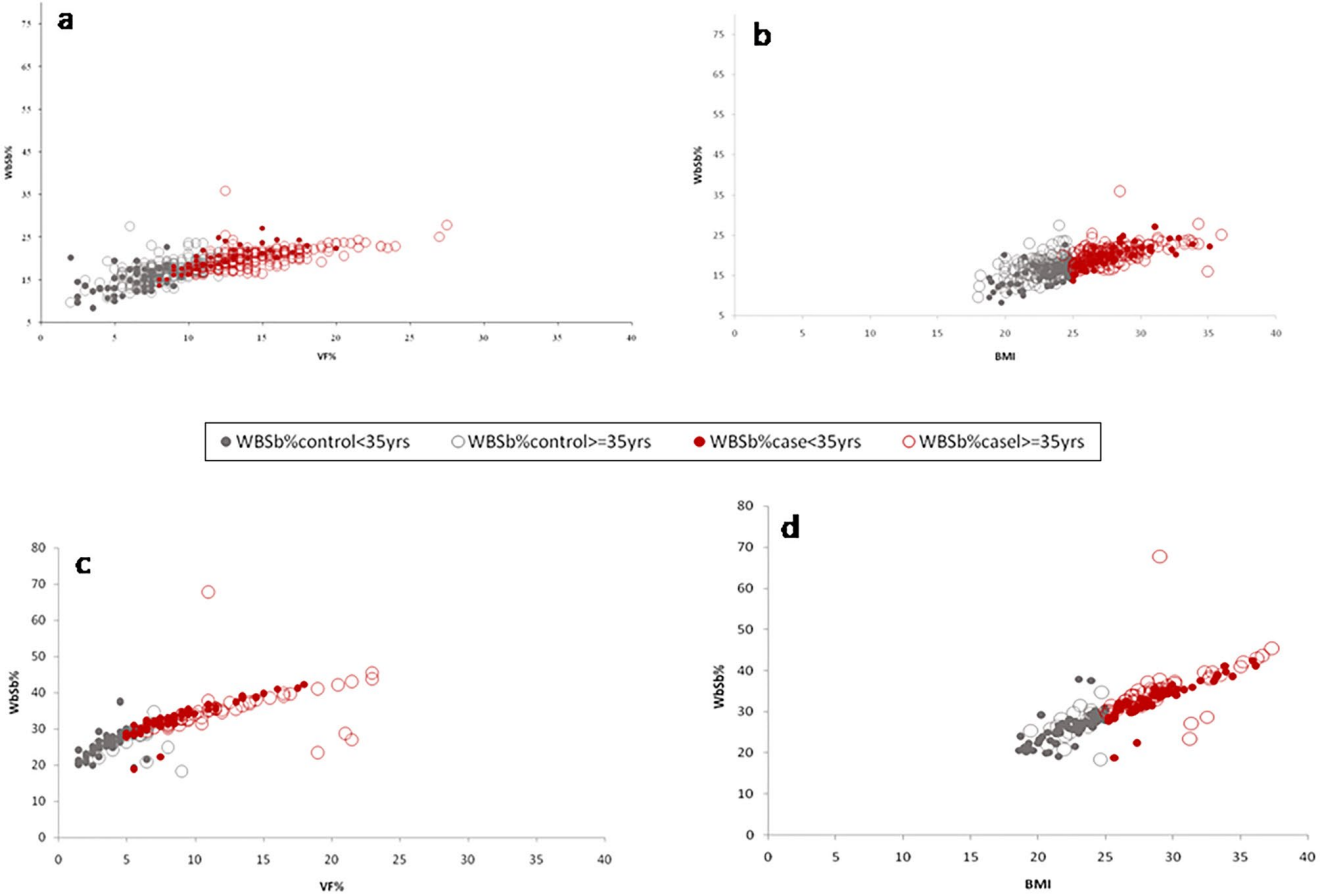
Association between (i) VF% and WbSb% (**a** & **c**) (ii) BMI and WbSb% (**b** & **d**) in the total male and female population respectively.

In the total male population, both VF% and BMI were found to be in statistically significant positive correlation with WbSb% but no common slope existed between them (Linear Regression Comparison: *F* = 11.68; *P* < 0.001) (Fig. 1a,b). In Group I, WbSb% was found to be positively correlated with VF% and BMI respectively in both the control (Linear Regression Comparison: *F* = 0.29; *P* = 0.58) and case groups (Linear Regression Comparison: *F* = 0.26; *P* = 0.6). In study Group II also, WbSb% was found to be positively correlated with VF% and BMI respectively in both the control (Linear Regression Comparison: *F* = 0.53; *P* = 0.46) and case groups (Linear Regression Comparison: *F* = 1.31; *P* = 0.25).

In the total female participants, VF% and BMI were both significantly positively associated with WbSb% (Linear Regression Comparison: *F* = 6.11; *P* < 0.01) (Fig. 1c,d). In Group I, a similar result was observed in both control (Linear Regression Comparison: *F* = 0.88; *P* = 0.35) and case groups (Linear Regression Comparison: *F* = 0.9; *P* = 0.34). In Group II females, although the control group reflected an absolutely different result with no significant association of WbSb% with VF% and BMI, here also both VF% and BMI exhibited identical patterns of action (Linear Regression Comparison: *F* = 0.97; *P* = 0.33). WbSb% showed a significant positive association with VF% and BMI in the case group (Linear Regression Comparison: *F* = 2.46; *P* = 0.12).

#### Comparative association between VF% and WbSk%; BMI and WbSk%

**Figure 2:**
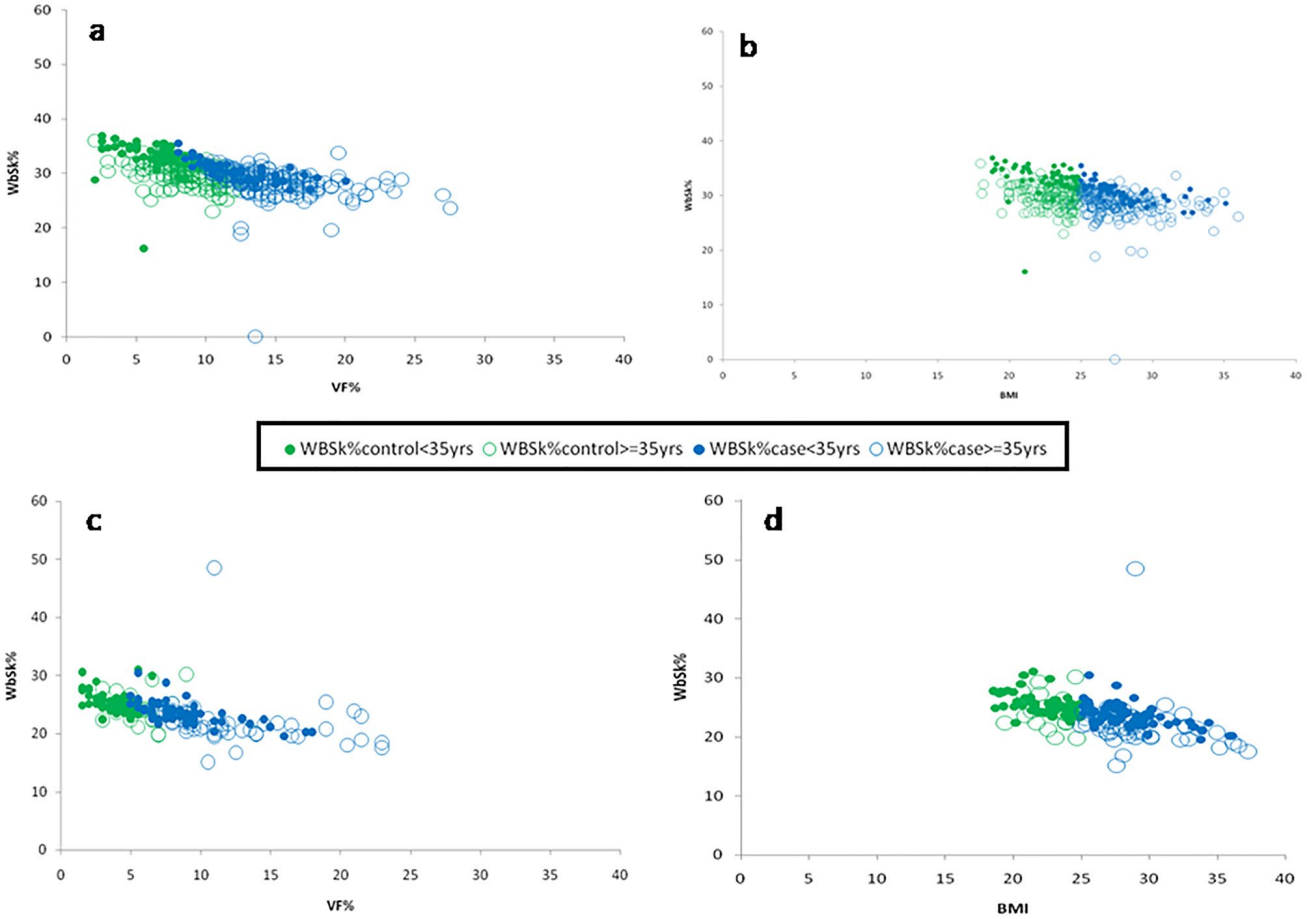
Association between (i) VF% and WbSk% (**a** & **c**) (ii) BMI and WbSk% (**b** & **d**) in the total male and female population respectively.

VF% and BMI were both significantly negatively correlated with WbSk% in both sexes. In the total male population, both VF% and BMI showed a similar negative correlation with WbSk% (Linear Regression Comparison: *F* = 0.35; *P* = 0.55) (Fig. 2a,b). In Group I male participants, WbSk% did not show any significant association with VF% or BMI in the control group (Linear Regression Comparison: *F* = 0.03; *P* = 0.85), contradictory to the result in the case group (Linear Regression Comparison: *F* = 0.004; *P* = 0.95). In Group II, WbSk% indicated a significant negative association with VF% and BMI in both control (Linear Regression Comparison: *F* = 0.69; *P* = 0.4) and the case groups (Linear Regression Comparison: *F* = 1.27; *P* = 0.25).

In the total female population also, VF% and BMI showed a comparable significant negative correlation with WbSk% (Linear Regression Comparison: *F* = 0.11; *P* = 0.73) (Fig. 2c,d). Both in control and case subgroups of Group I, WbSk% were significantly negatively correlated with VF% and BMI (Linear Regression Comparison: *F* = 0.03, *P* = 0.85; Linear Regression Comparison: *F* = 0.003, *P* = 0.95). In contrast, the control and case females of Group II did not show any significant correlation (Linear Regression Comparison: *F* = 0.11, *P* = 0.74; Linear Regression Comparison: *F* = 0.06; *P* = 0.78). Common slopes between VF% with WbSk% and BMI with WbSk% linear regression curves were present in all the groups irrespective of age and sex.

#### Association of VF% and BMI with the blood pressure parameters (DBP and MAP)

**Figure 3:**
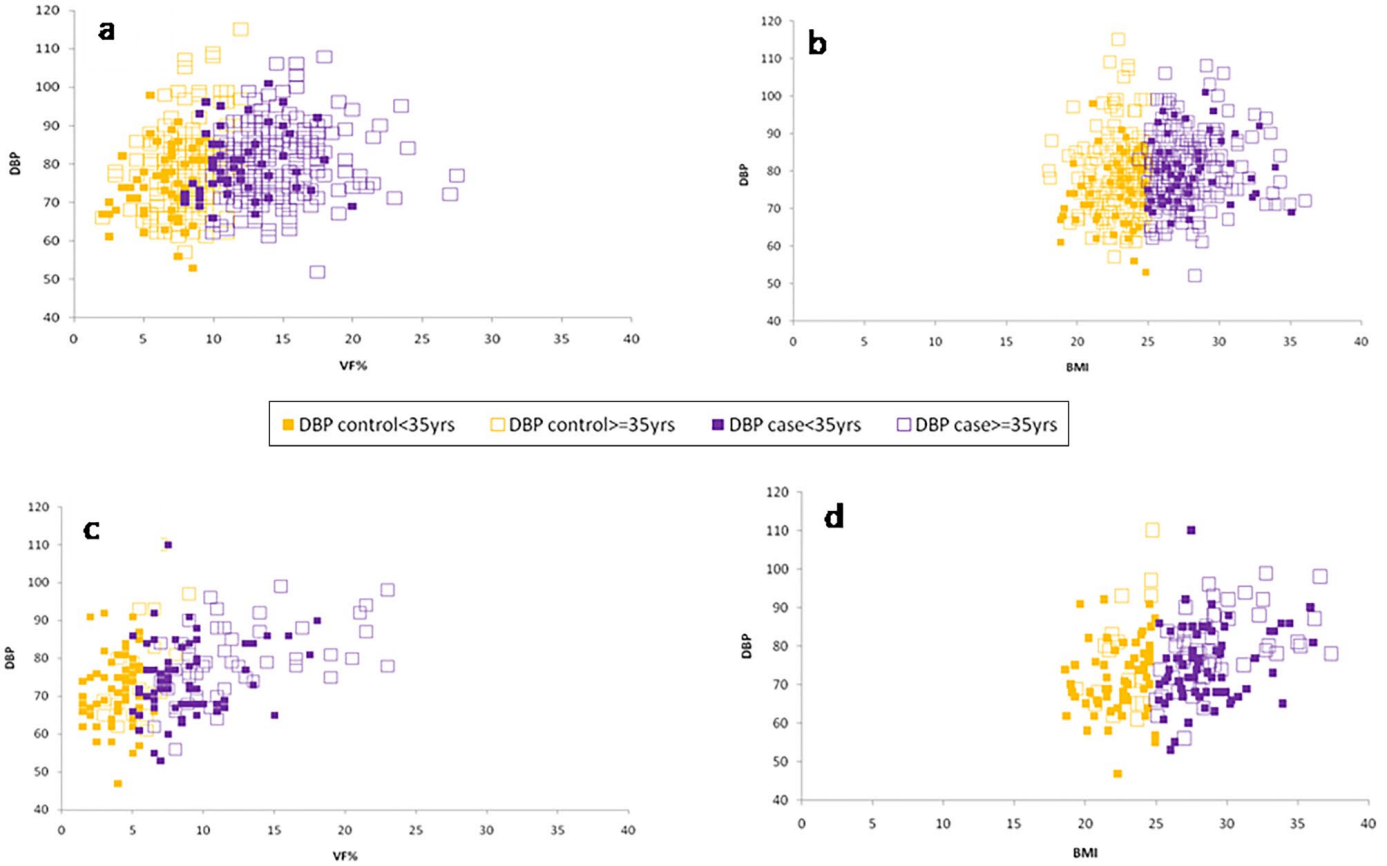
Association between (i) VF% and DBP (**a** & **c**) (ii) BMI and DBP (**b** & **d**) in the total male and female population respectively.

**Figure 4:**
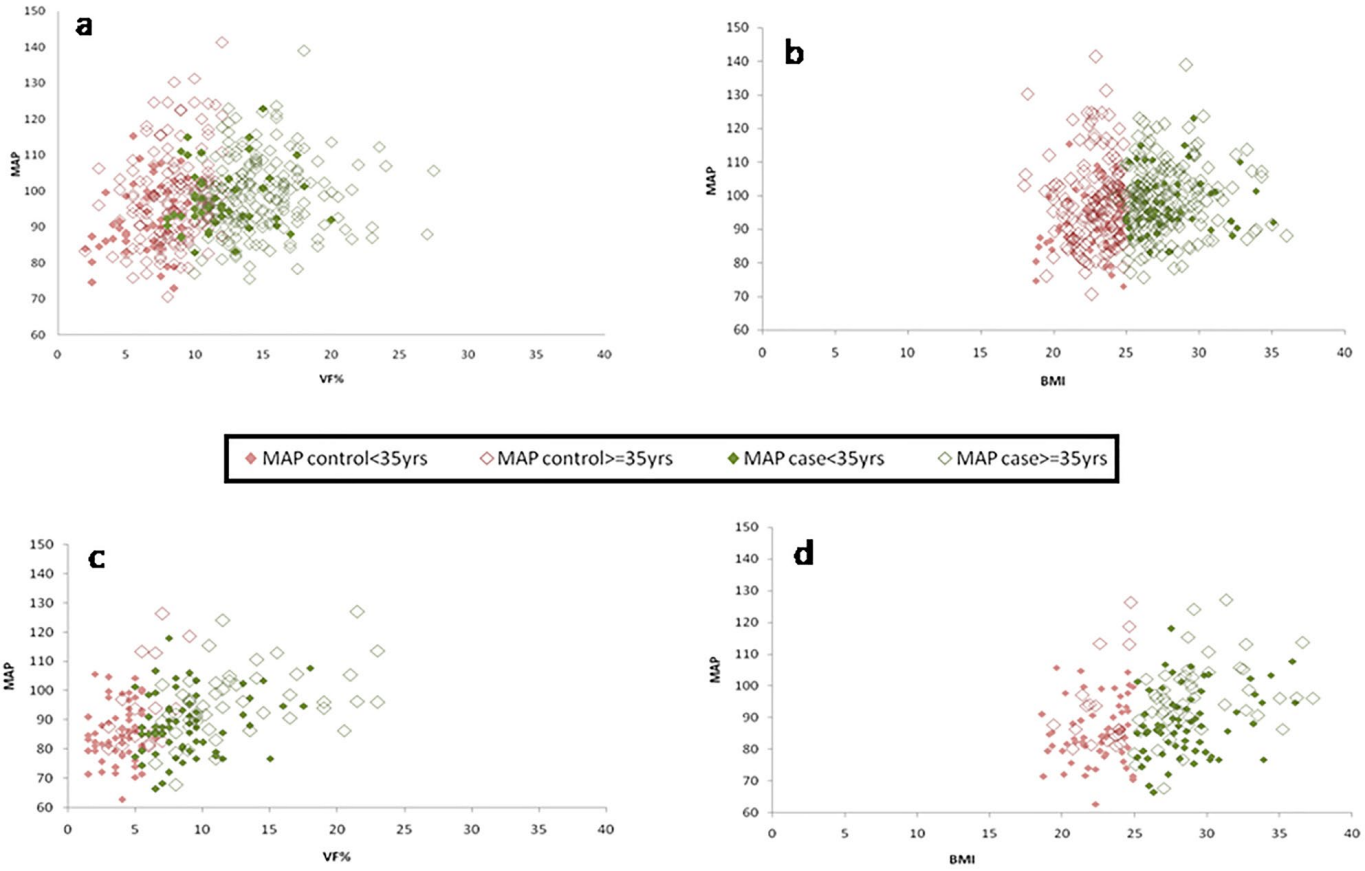
Association between (i) VF% and MAP (**a** & **c**) (ii) BMI and MAP (**b** & **d**) in the total male and female population respectively.

SBP association with VF% and BMI indicated gender biases. It was not significantly positively correlated in males (Linear Regression Comparison: *F* = 0.13, *P* = 0.71) (Supplementary Fig. 2a,b), but it was in the female group (Linear Regression Comparison: *F* = 0.51, *P* = 0.47) (Supplementary Fig. 2c,d). In the total male population, DBP and MAP were both significantly positively correlated with VF% and BMI (DBP: Linear Regression Comparison: *F* = 0.09, *P* = 0.75; MAP: Linear Regression Comparison: *F* = 0.00, *P* = 0.99) separately (Figs. 3a,b, 4a,b). In the total female population, DBP and MAP were both significantly positively correlated with VF% and BMI (DBP: Linear Regression Comparison: *F* = 0.003, *P* = 0.95; MAP: Linear Regression Comparison: *F* = 0.14, *P* = 0.7) respectively (Figs. 3c,d, 4c,d). The overweight and obese females in both Group I and Group II showed a significant positive correlation of both DBP and MAP with BMI and VF% (Supplementary Table 2).

### Questionnaire-based analysis

The parameters studied using structured questionnaire-based analysis represented a significant impact on obesity-related health outcomes.

#### Working and sleeping durations were significantly different among individuals of the subgroups in the study population

There were significant differences observed among Group I as well as Group II participants (Working hours: *T* = 5.84, *DF*_*1,2*_ = 229,415, *P* < 0.001; Sleeping hours: *T* = 6.95, *DF*_*1,2*_ = 229,415, *P* < 0.001) (Supplementary Table 1b).

#### Physical activity is not a significant factor associated with decreasing VF% and BMI in our population

Physical activity showed no significant correlation with VF% and BMI in the total study participants (n = 559) (Supplementary Fig. 3a–d).

#### Presence of parental history of obesity, CVD, T2DM, and hypothyroidism significantly affects the occurrence of obesity in the offspring

**Figure 5:**
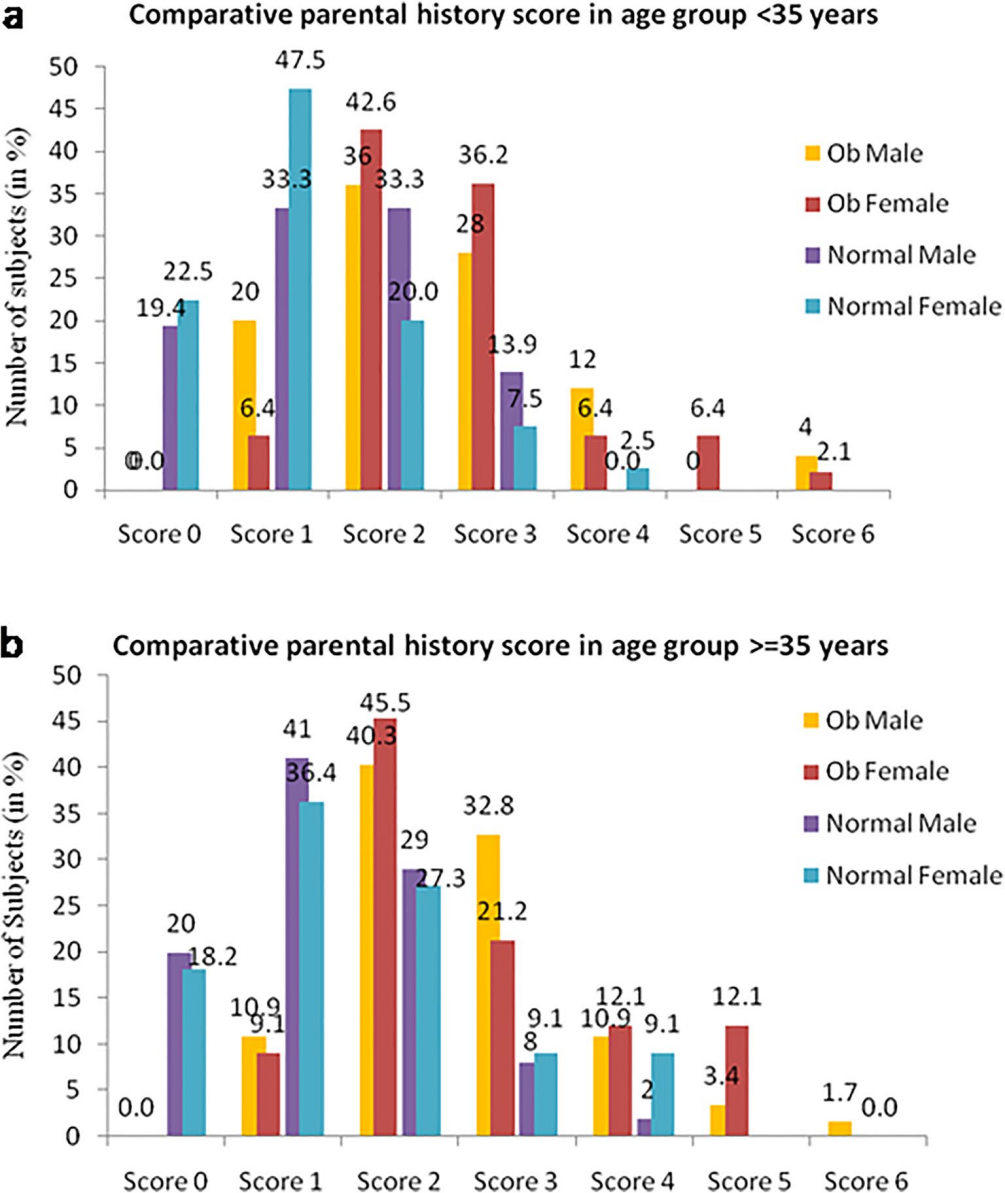
Comparative parental history score in different subgroups of Group I (**a**) and Group II (**b**) of the studied population.

The data scoring of the parental history of mentioned diseases in 411 study participants indicated a significant positive correlation between the case and control groups of both the sexes (*T* = 12.81, *DF*_1,2_ = 223,186, *P* < 0.001). Increasing percentages of individuals with parental history of the concerned diseases were observed in all the case groups, irrespective of sexes (Fig. 5a,b). The score analysis could thereby predict that the parental history of the disease can significantly affect the offspring’s health, whereas score 2 is the transition score between the lower and higher risk of parental disease inheritance.

#### The presence of obesity-associated co-morbidities in the population

The results of the analyses identified the presence of T2DM in the Group I case individuals with higher incidents in Group II. Hypothyroidism expressed a sex-biased pattern of occurrence in females of both groups. The initial manifestation of arthritis was also observed in case individuals of Group I, increasing from ten to fifteen folds with age. The rate of hypertension among the Group I case males (33%) and females (17%) established a predominant risk of CVD with their increasing age (Supplementary Fig. 4).

### Blood biochemical analysis

Analyses of the biochemical parameters (Supplementary Fig. 5) from the selected 45 case individuals supported the presence of 25-OH Vit D deficiency in 65% of the studied samples. Significant anomalies in liver function tests and complete blood count were also observed.

## Discussion

Recent studies have indicated that South Asian populations have a higher tendency of increasing obesity-linked non-communicable diseases as compared to Caucasians^28^. These findings along with insufficient information on obesity-associated non-communicable diseases in a varied age group have aroused our interest to study a South-East Indian adult population. In our study, we observed significant differences in both the BMI and VF% between the sexes. In the studied diversified age groups, VF% and BMI both showed a significant positive correlation with WbSb% and a negative correlation with WbSk% irrespective of individual obesity status. The control females of Group II indicated an entirely different result, but it could be considered an error due to a very low sample size. Therefore, as per our observation, VF% exhibited similar relation with fat deposition (WbSb%) and skeletal muscle mass (WbSk%) as in the case of BMI. Thus, VF% along with BMI portrayed a more authentic way to diagnose the lifestyle disease predisposition. The absence of any correlation between both BMI and VF% with WbSk% in Group I control males and Group II control females is a point of consideration. Young men without obesity have a higher lean skeletal muscle mass as compared to the overweight and obese individuals of the same age and sex-matched group. Both fat deposition and muscle loss are minimal in this group. Increased sarcopenia, i.e., lower WbSk% with higher VF% was observed in Group II male individuals. Earlier studies may have an answer to this observation, as sarcopenia increases both with age and lifestyle diseases like obesity^29^. Moreover, elder men with obesity are highly susceptible to sarcopenia due to hypogonadism^30^.

Cardiovascular diseases (CVD) including coronary heart diseases (CHD), heart failure (HF), hypertension (HT), and arrhythmias are the leading cause of global death^31^. About one-quarter of the adult population in the world is hypertensive and by 2025 the proportion would increase to 29%^32,33^. CVD accounted for 15–20% of all deaths in the Indian subcontinent^34^. It was seen in several studies that the prevalence of hypertension was more in females (52.5%) as compared to males (27.3%)^35^. A recent study on women showed a high prevalence of hypertension with the strongest association with overweight and obesity^36^. Our study results reflected obesity to be a potential allied factor for increasing blood pressure parameters only in females with overweight and obesity, of both age groups. But more precisely, it could be said that central obesity (VF%) affected hypertension in only case women of Group II. Moreover, hypertension was more predominant in the Group I case individuals, than in the control ones, indicating the tendency to develop CVD to be more among the young case subjects. Thereby, controlling obesity from early childhood may be a preventive measure to decrease the risk of developing CVD at an older age.

It has been recorded that T2DM in India has already affected 77 million in 2020^37^. T2DM is significantly present in Group II irrespective of sex, however, the Group I case individuals also reported the disease incidences. The prevailing risk of T2DM was thereby found to be influenced by increased age and enhanced by obesity. In the case of hypothyroidism, both obesity and sex biases reflected their role in the occurrence of disease among case females of all age groups. Arthritis, on the other hand, showed only an age-dependent pattern, increasing prominently in Group II individuals.

Sedentary behavior along with a lack of moderate-vigorous physical activity is negatively associated with obesity-related co-morbidities^38^. A 150–300 min of moderate or 75–150 min of vigorous aerobic physical activity per day is considered to be ideal for a healthy lifestyle in adults up to 64 years of age^39^. Automation and digitization have increased physical inactivity by several folds^40^. A sedentary work pattern in information technology professionals, similar to our study population, should have reflected a similar scenario. Yet in a study, only 16% were found to have increased adiposity^41^. The installation of fitness equipment and healthcare facilities in such multinational companies might be the game-changer there. Analysis of our data revealed physical activity as an insignificant factor to be considered in our study as all the participants irrespective of age and body composition were not even engaged in moderate aerobic exercises. Despite being aware of their health, they lacked the motivation for physical activity as well as easily accessible physical workout facilities and were comfortable in a sedentary lifestyle with very minimal physical movement, where the most physically active group showed Mean = 203.7 min of physical activities per week, the Mode being 0 (Group I control males). The increasing pattern of obesity thus becomes difficult to control.

The significance of the parental history of obesity and related co-morbidities alongside environmental effects has always been inevitable in studying the disease risk in the offspring^42^. Several studies have confirmed this association in children and adolescents^18,42^, yet its long-term effects have been left unnoticed in the adult population. This purpose was addressed in our study. The parental history of obesity-associated heritable diseases was found significantly higher in the case group of our studied population irrespective of age and sex. A complex gene-environment interaction thereby indicates the disease predisposition.

Energy intake, although an inevitable phenomenon, is often irregular and uncontrolled in different individuals. In Kolkata, West Bengal, a study reported employees with a sedentary lifestyle have equivalent energy intake to that of physically active laborers, thereby resulting in significantly higher body weight^43^. In the Indian population, energy intake is restricted to 39 kcal/kg body weight/day in men and 35 kcal/kg body weight/day in women with a sedentary lifestyle^44^. Indian diets have gradually become more westernized, influenced by a multitude of factors such as rising income, demographic transition, urbanization, and the spread of retail chains or supermarkets^45^. In the current years, expenses for staple cereal consumption in urban India have decreased to 6.6% of the total expenses for food whereas expenses for processed and protein-rich food have increased to 30%^46^. In our studied case population with a similar urbanized food habit, the blood profiling reflected certain distinct parameters to be significantly associated with increased BMI. Vitamin D deficiency, as observed in 65% of our case individuals, may be responsible primarily for increased bone turnover, increased fracture risk, and secondarily for other metabolic and autoimmune disorders, even cancer^47^. The lack of adverse effects on bone in obese individuals may indicate that serum 25(OH)D is low due to volumetric dilution as the adipose tissue acts as the reservoir of vitamin D^48^. In individuals with cardiovascular disease risk, vitamin D deficiency was found to be associated with a decrease in high-density lipoprotein (HDL) concentration and an increase in low-density lipoprotein (LDL) concentration. It triggered inflammation both in epicardial fat and in the vascular walls thereby increasing vascular rigidity^49^. Excess fat accumulation also affects the liver’s functioning as it is the major organ controlling fat metabolism. Among our studied parameters, serum glutamic-pyruvic transaminase (SGPT) was observed to be high in 55.8% of individuals which may indicate the onset of non-alcoholic fatty liver disease (NAFLD)^50^. Although it is believed that the RBC count increases with increasing physical activity^51^, in our study high RBC count was observed in 66.67% of the population. An increased level of RBC count may be an indicator of developing metabolic diseases, as observed in the Iranian population^52^. An improper diet with insufficient nutrients may contribute to the development of obesity-associated metabolic disease risk.

Our study had certain limitations as well. The population distribution was randomized, hence sex biases were unavoidable (male and female staffs are in the ratio are 2:1)^53^. It is noteworthy to mention here that information regarding physical activity, diet, etc. in the questionnaire was recorded as per our study participants’ statements.

Parental history of obesity-related co-morbidities, as evident in our study, results in longitudinal transmission. Moreover, a sedentary lifestyle amplifies it several folds. In our studied population, the obesity rate although similar in Group I and Group II individuals, the co-morbidity effects express more in case participants in Group II, women being the vulnerable clan. The current study observations can be utilized for pathophysiological implementation of diagnostic techniques involving screening of obesity in adolescence. Health care programs, incorporation of physical fitness activities in academic houses, awareness and motivation including a prescribed diet, balanced lifestyle with sufficient physical activity, and regular monitoring of the VF% alongside BMI will provide satisfactory results in the long run. Early diagnosis and control of obesity through school and higher academic-based health-planning programs is one of the most effective measures to curb the growing graph of global obesity.

## Supplementary Information

Supplementary Information.

## Data Availability

The datasets generated during and/or analysed during the current study are available from the corresponding author on reasonable request.
