# Supplementary material for: Sedentary lifestyle with increased risk of obesity in urban adult academic professionals: an epidemiological study in West Bengal, India
Source: Sci Rep. 2023 Mar 25;13:4895. doi: 10.1038/s41598-023-31977-y (PMC10039938; doi:10.1038/s41598-023-31977-y)
Supplement: Supplementary file 1 — Supplementary Information. [file 41598_2023_31977_MOESM1_ESM.pdf]

Sedentary Lifestyle with Increased Risk of Obesity in Urban Adult Academic Professionals: An Epidemiological Study in West Bengal, India.

Sunandini Ghosh[a,b], Manabi Paul[a], Kousik Kumar Mondal[c], Sandip Bhattacharjee[d] and Pritha Bhattacharjee[a#]

[a]Department of Environmental Science, University of Calcutta, 35, Ballygunge Circular Road, Kolkata 700019, West Bengal, India
[b] Department of Zoology, University of Calcutta, 35, Ballygunge Circular Road, Kolkata 700019, West Bengal, India
[c] Department of Zoology, Mugberia Gangadhar Mahavidyalaya, Bhupati Nagar, Purba Medinipur 721425, West Bengal, India
[d] Health Management, Siemens Limited, Mumbai, India
#corresponding author
PrithaBhattacharjee, PhD
Environmental Epigenomics Lab,
Department of Environmental Science,
University of Calcutta,
35, Ballygunge Circular Road, Kolkata 700019, West Bengal,India
Phone: +91-9432910619
Email: 777.pritha@gmail.com, pbenvs@caluniv.ac.in

Supplementary Fig. 1

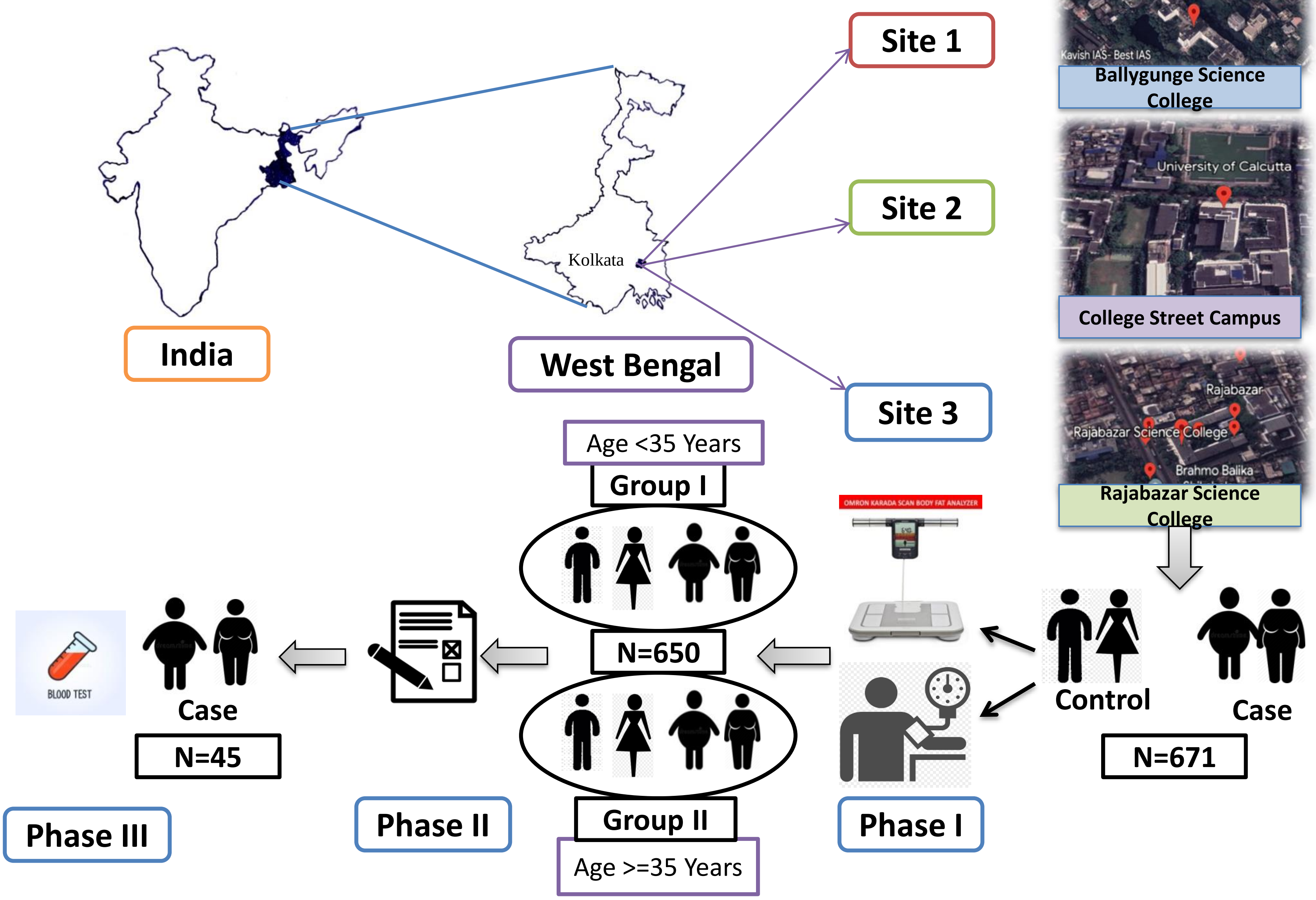

Schematic representation of the methodology

Supplementary Table 1a: Study group age and frequency

Grouping criteria of the study population					
**Age**	**Sex**		**Frequency n (%)**	**Mean Age ± SD**	**Mean BMI ± SD**
**<35 years (n= 232)**	Male	Case	51 (7.8%)	26.44±4.04	28.04±2.44
		Control	56 (8.6%)		22.61±1.82
	Female	Case	65 (10%)	24.44±3.22	28.63±2.65
		Control	60 (9.2%)		22.39±1.84
**>=35 years (n= 418)**	Male	Case	190 (29.2%)	48.38±7.27	27.75±2.24
		Control	159 (24.4%)		22.83±1.49
	Female	Case	50 (7.7%)	46±6.58	29.24±3.13
		Control	19 (2.9%)		22.78±1.51

Supplementary Table 1b: Socio-demographic details of the study population (n=650)

Socio-demographic characteristics	Sub categories		Frequency n (%)
**Addiction**	Smoking	Male	139 (21.41%)
		Female	1 (0.15%)
	Chewing	Male	12 (1.8%)
		Female	0 (0)
	Alcohol	Male	79 (12.17%)
		Female	1 (0.15%)
**Food Preference**		Non-Vegetarian	627 (96.31%)
		Vegetarian	19 (2.9%)
			**Mean ± SD**
**Daily Water Consumption(L)**			3.17± 1.18
**Working Hours**	Age <35 years		10.11± 1.73
	Age >=35 years		7.65± 1.89
**Sleeping Hours**	Age <35 years		6.13± 1.46
	Age >=35 years		6.49± 1.18

Supplementary Table 2: Regression Analyses Results

Parameter 1	Sex	Parameter 2	Linear Regression (R^2)	Std Beta	P Value
**WbSb%**	Male	VF%	0.54	0.73	**<0.001**
		BMI	0.49	0.7	**<0.001**
	Female	VF%	0.51	0.71	**<0.001**
		BMI	0.63	0.79	**<0.001**
**WbSk%**	Male	VF%	0.32	-0.57	**<0.001**
		BMI	0.16	-0.4	**<0.001**
	Female	VF%	0.25	-0.5	**<0.001**
		BMI	0.23	-0.48	**<0.001**
**SBP**	Male	VF%	0.009	0.09	0.048
		BMI	0.002	0.04	0.29
	Female	VF%	0.13	0.36	**<0.001**
		BMI	0.06	0.26	**<0.001**
**DBP**	Male	VF%	0.02	0.16	**<0.001**
		BMI	0.02	0.14	**<0.005**
	Female	VF%	0.14	0.37	**<0.001**
		BMI	0.11	0.33	**<0.001**
**MAP**	Male	VF%	0.02	0.14	**<0.005**
		BMI	0.01	0.11	**<0.05**
	Female	VF%	0.16	0.4	**<0.001**
		BMI	0.1	0.32	**<0.001**
**PA**	Male	VF%	0.008	-0.089	0.075
		BMI	0.007	-0.081	0.1
	Female	VF%	0.02	-0.16	0.047
		BMI	0.03	-0.18	**<0.05**

Supplementary Table 3: Biochemical Parameters Analyzed from Blood

	Sl. No.	Name of Test	Reference Range
**Glucose Profile**	1	Fasting Blood Sugar	70-105 mg/dl
	2	Average Blood Sugar	90-180 mg/dl
	3	HbA1C	<6-<8%
**Ion Profile**	4	Vitamin D	<20 ng/ml
	5	Vitamin B	211-911 pg/ml
	6	Calcium (Ca)	8.8- 10.6 mg/dl
	7	Iron (Fe)	Male= 65-175, Female= 50-170 µg/dl
	8	Fe binding	Male= 225- 535, Female= 215- 535 µg/dl
	9	Transferrin %	13-45
**Lipid Profile**	10	Total Cholesterol	125- 200 md/dl
	11	HDL	35-80 mg/dl
	12	LDL	85- 130 mg/dl
	13	Triglycerides	25- 200 mg/dl
	14	VLDL	5- 40 mg/dl
**Renal Profile**	15	Uric Acid	3.1- 7.8 mg/dl
	16	Serum Creatinine	0.5- 0.8 mg/dl
	17	Protein	5.7- 8.2 gm/dl
	18	Serum Albumin	3.2- 4.8 gm/dl
	19	Serum Globulin	2.5- 3.4 gm/dl
**Thyroid Profile**	20	T3	60-180 ng/dl or 0.9-2.8 nmol/L
	21	T4	4.5- 12 µg/dl
	22	TSH	0.3- 5.5 µIU/ml
**Liver Profile**	23	Alkaline phosphatase	42-98 U/L
	24	Billirubin	0.3- 1.2 mg/dl
	25	GGT	<38 U/L
	26	SGOT	<40 U/L
	27	SGPT	13-40 U/L
**Complete Blood Count**	28	Total Leukocytes	4-10 X 10^3 / µL
	29	Neutrophils	40-80%
	30	Lymphocytes	20-40%
	31	Monocytes	0-10%
	32	Eosinophils	0-6%
	33	Basophils	<2%
	34	Absolute Neutrophil	2-7 X 10^3 / µL
	35	Absolute Lymphocytes	1-3 X 10^3 / µL
	36	Absolute Monocytes	1-2 X 10^3 / µL
	37	Absolute Eosilophils	0.02-0.5 X 10^3 / µL
	38	Absolute Basophils	0.02-0.1 X 10^3 / µL
	39	Total RBC	Male=4.7- 6.1, Female=4.2-5.4 X 10^6/µL
	40	Heamoglobin	12-17 gm/dl
	41	PCV	36-46%
	42	MCV	80-101fL
	43	MCH	27-32pQ
	44	MCHC	31.5-34.5 g/dl
	45	RDW-CV	11.6-14%
	46	PDW	9.6-15.2fL
	47	MPV	6.5-12fL
	48	Platelet Count	150-400x 10^3 / µL

Supplementary Fig. 2

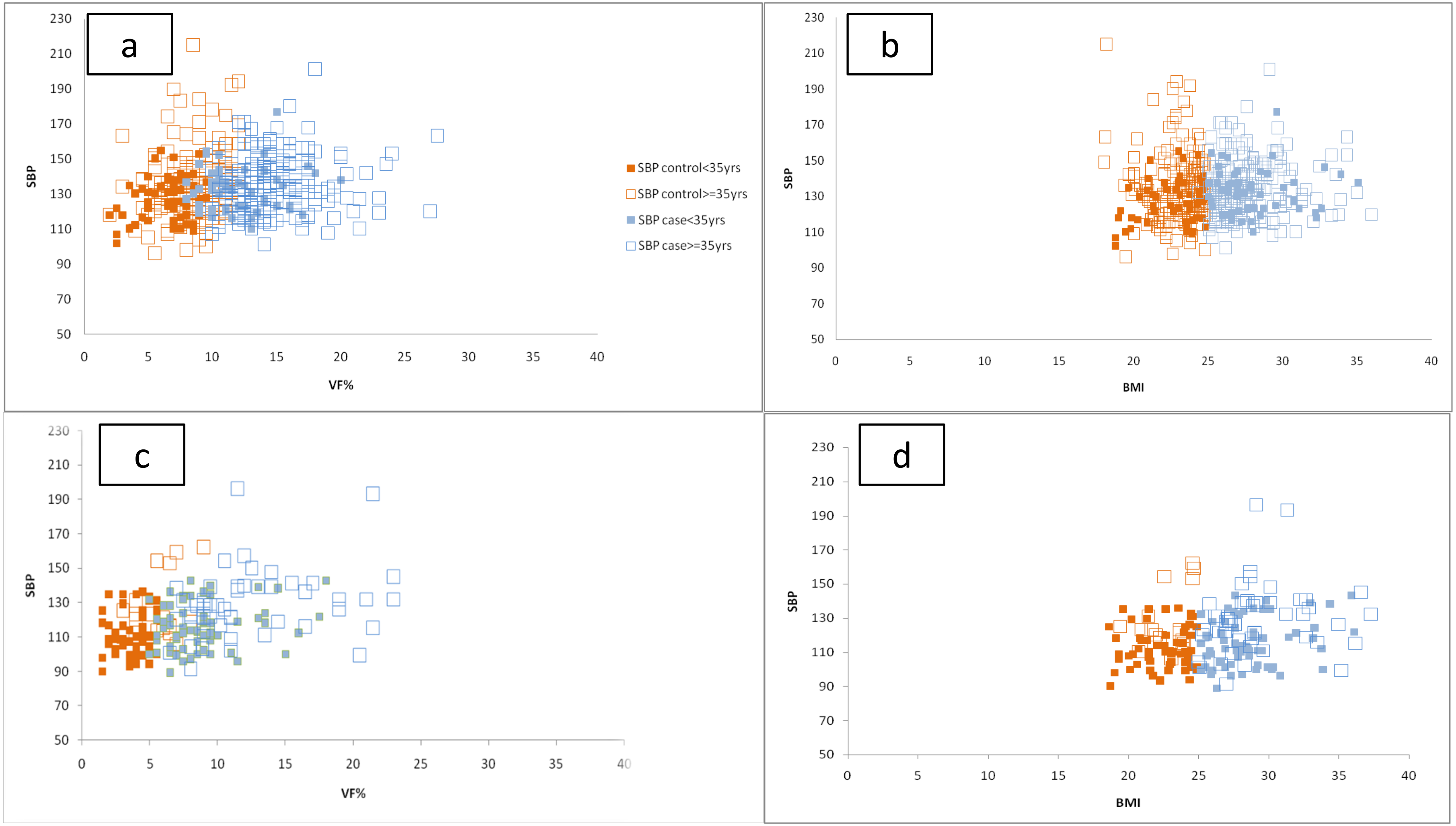

Association between VF% and SBP in the total male population (a) and female population (c)

Association between BMI and SBP in the total male population (b) and female population (d)

Supplementary Fig. 3

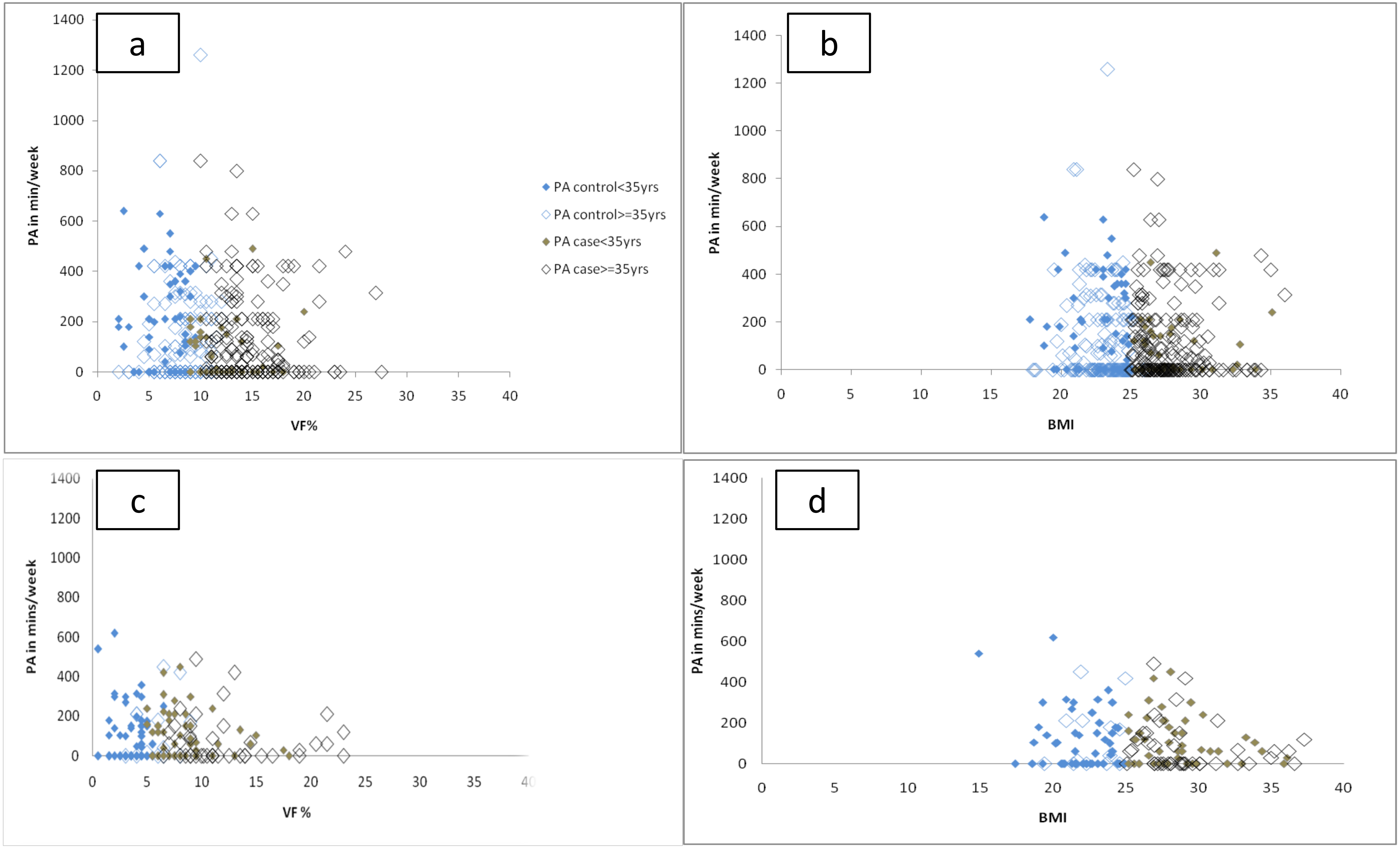

Association between VF% and PA in the total male population (a) and female population (c)
Association between BMI and PA in the total male population (b) and female population (d)

Supplementary Fig. 4

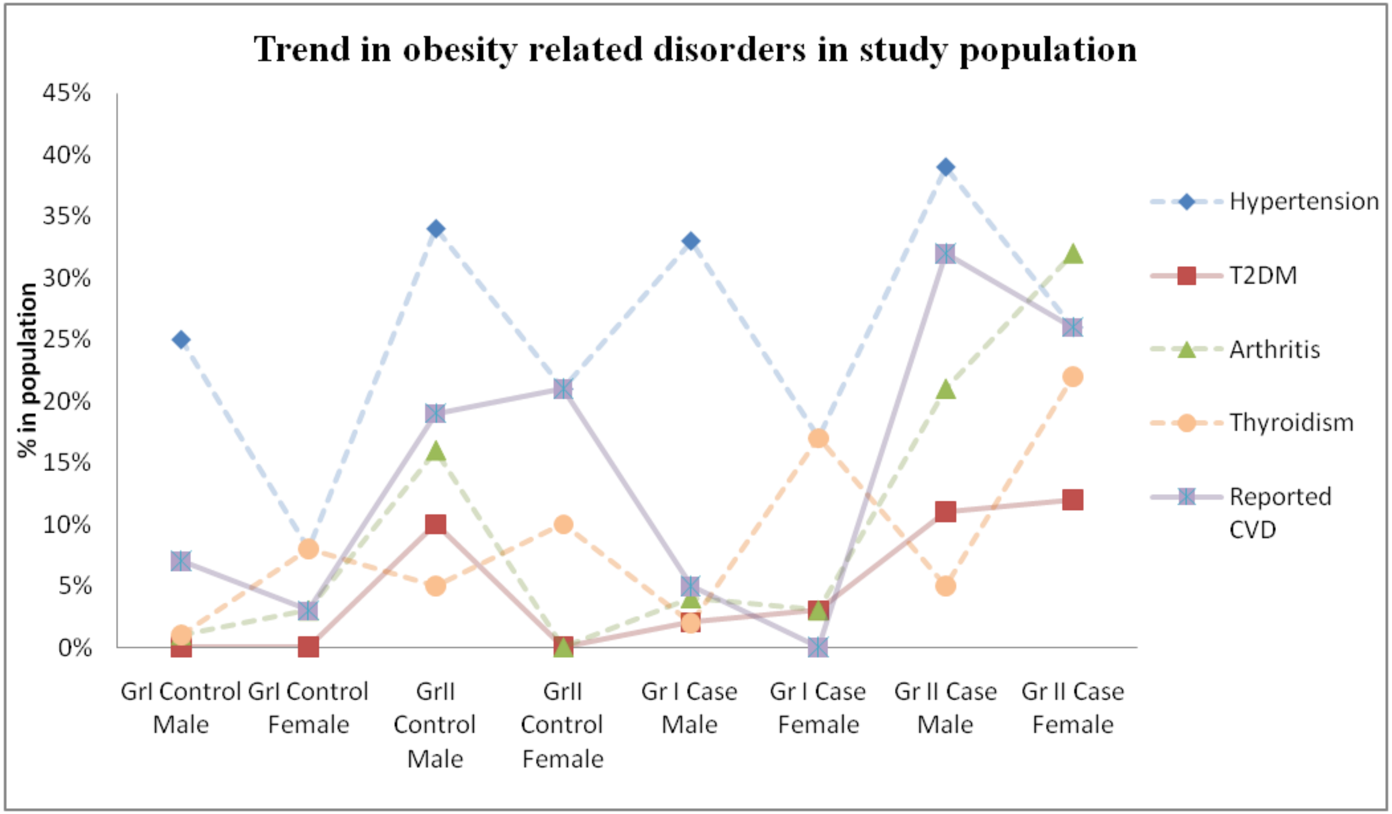

Trend in obesity associated co-morbidities among all the subgroups of the study population.

Supplementary Fig. 5

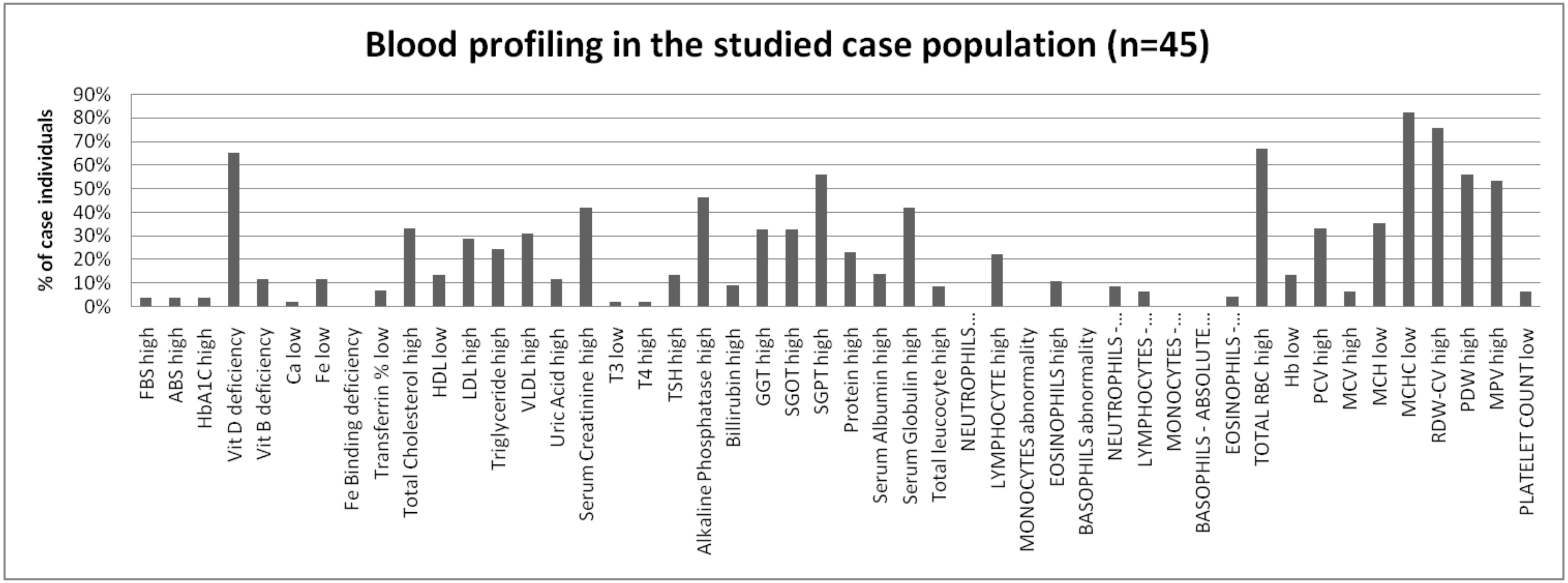

Blood profile parameters in the studied population (n=45)

Effect size Calculations for unpaired 2-tailed t-Test

Sample size: Male (n=456)
Female (n=194)

**Parameter**	**Cohen's *d***	**Glass's *delta***	**Hedges'*g***
VF%	0.774	0.800	0.784
BMI	0.234	0.264	0.245
WbSb%	2.610	3.912	2.964
WbSk%	2.093	2.313	2.174
SBP	0.917	0.922	0.919
DBP	0.396	0.404	0.399
MAP	0.689	0.709	0.696

Calculation site:
https://www.socscistatistics.com/effectsize/default3.aspx

References for Supplementary Fig. 1

1. Site 1 Map: https://earth.google.com/web/search/Ballygunge+Science+College,+University+Of+Calcutta,+Ballygunge+Circular+Road,+Ballygunge,+Kolkata,+West+Bengal/@22.5273218,88.3627717,12.73358625a,966.99768346d,35y,0h,45t,0r/data=CtsBGrABEqkBCiUweDNhMDI3NzI4MWQwYmU4MTU6MHg0YWI2ZTQ0NTM2NTUyMWY1GcV3vY_-hjZAIYTayqY3F1ZAKm5CYWxseWd1bmdlIFNjaWVuY2UgQ29sbGVnZSwgVW5pdmVyc2l0eSBPZiBDYWxjdXR0YSwgQmFsbHlndW5nZSBDaXJjdWxhciBSb2FkLCBCYWxseWd1bmdlLCBLb2xrYXRhLCBXZXN0IEJlbmdhbBgCIAEiJgokCYEn8Za3lDZAERDzyN16kjZAGQuEbOXTF1ZAITYJIDqgFlZAKAI
2. Site 2 Map: https://earth.google.com/web/search/University+of+Calcutta,+College+Street,+Calcutta+University,+College+Square,+Kolkata,+West+Bengal/@22.5750862,88.3629188,10.2682303a,966.6255932d,35y,0h,45t,0r/data=Cs4BGqMBEpwBCiUweDNhMDI3N2FkMjkzYjVmY2Y6MHg1NWMzMmFjNmFjMDE1NTNkGYNhZdk4kzZAIaFGxg86F1ZAKmFVbml2ZXJzaXR5IG9mIENhbGN1dHRhLCBDb2xsZWdlIFN0cmVldCwgQ2FsY3V0dGEgVW5pdmVyc2l0eSwgQ29sbGVnZSBTcXVhcmUsIEtvbGthdGEsIFdlc3QgQmVuZ2FsGAIgASImCiQJALJ4FM-WNkARPEfyItuSNkAZOyZV30MYVkAhugXASjAXVkA
3. Site 3 Map: https://earth.google.com/web/search/rajabazar+science+college/@22.529886,88.360196,7.7862875a,5164.70121125d,35y,0h,0t,0r/data=CigiJgokCQAbNL-uiTZAEVwqSnWehTZAGSm1qM6NF1ZAIZ-lzBeNFlZA
